# Quality of life of transplanted children and their parents: a cross-sectional study

**DOI:** 10.1186/s13023-021-01987-y

**Published:** 2021-08-17

**Authors:** Pauline Duvant, Magali Fillat, Florentine Garaix, Bertrand Roquelaure, Caroline Ovaert, Virginie Fouilloux, Michel Tsimaratos, Pascal Auquier, Alexandre Fabre, Karine Baumstarck

**Affiliations:** 1 Service de pédiatrie multidisciplinaire, Hôpital de la Timone, Marseille, France; 2 Service médico-chirurgical de cardiologie pédiatrique et congénitale, Hôpital de la Timone, Marseille, France; 3 EA 3279 CEReSS - Health Service Research and Quality of Life Center, Aix-Marseille Univ, 27 bd Jean Moulin, 13385 Marseille Cedex 05, France

**Keywords:** Transplant organ, Paediatric, Children, Quality of life, Parents

## Abstract

**Background:**

Transplantation is a saving therapeutic that has heavy consequences. The quality of life (QoL) of transplanted children and their parents has been little studied and should help physicians better manage these patients. The objectives of the study were to assess: (1) the QoL of transplanted children and parents and compare it with that of children with other chronic conditions associated with long-term consequences, and (2) potential variables modulating the QoL.

**Methods:**

This cross-sectional study was performed in a multidisciplinary paediatric unit (Timone Hospital, Marseille, France). Children were less than 18 years old; had a liver, kidney or heart transplant; and had a time since transplantation of 1–10 years. Socio-demographics and clinical data were recorded from medical forms. The QoL was assessed using the VSP-A (Vécu et Santé Perçue de l’Adolescent et de l’Enfant) and the WhoQoL self-reported questionnaires.

**Results:**

Forty-five families were included (response rate: 76%). The transplanted organs were the liver for 20 children, the kidney for 15 children, and the heart for 10 children. The QoL of transplanted children reported by their parents was better than that of children with inborn errors of metabolism and similar to that of childhood leukaemia survivors. The QoL of parents of transplanted children was better than that of parents of children with inborn errors of metabolism and did not differ from French norms. The QoL did not differ according to the nature of the transplanted organ, sex or the main sociodemographic data. The main modulators decreasing QoL were residual treatment level, medications switch and the presence of another regular treatment.

**Conclusion:**

Transplanted children and their families reported a fairly preserved QoL compared to children with other chronic health conditions. Special attention should be given to QoL modulators related to therapeutic management (medication switches, regular treatments) that might be amenable to improve the QoL.

***Trial registration*** Ethics committee of Aix-Marseille University, France (reference number: 2014-08-04-03, 24/4/2015; https://www.univ-amu.fr/fr/public/comite-dethique).

**Supplementary Information:**

The online version contains supplementary material available at 10.1186/s13023-021-01987-y.

## Background

Paediatric organ transplantation is now a standard treatment for end-stage disease. The survival rate afterwards transplantation may be up to 80% 5 years after [1–4]. Transplantation is not a curative treatment and after, the ongoing chronic illness remains present, with daily immunosuppressive treatment and potential unpleasant side effects, fear of organ dysfunction, and the need for continuous medical supervision [5]. Organ transplantation for children, due to its occurrence during physical and mental development, causes major lifestyle disruptions in the everyday lives of patients and their families [6, 7] impacting their quality of life (QoL) [8].

The study of the QoL of transplanted children and their parents and the identification pf potential factors of QoL modulation should help to better manage these populations. Among the studies exploring the QoL of transplanted children, the findings are conflicting. In comparison with healthy populations, the QoL of transplanted paediatric patients could be lower [2, 5, 9–13], closer or higher [5, 9, 14–16], with children’s QoL often being lower and adolescents’ QoL being higher. Compared to other chronic conditions, the results are also discordant, with some studies showing better [17, 19], similar [13, 18, 19] or lower QoL [11]. Three studies in the literature explored the QoL of parents of transplanted children [11, 15, 20]: they show deteriorated QoL in comparison with the general population [15] and more mental health problems [11]. Most of these studies analysed the transplantations of one organ type, liver or kidney, which limits the practical application of the results due to a low number of patients. Indeed, organ transplantation is rare, and transplantation centers have only a few patients. In our hospital in Marseille, France, all heart, liver and kidney transplanted children are followed up in the same unit and are taking care of by the same team. Follow-up and the management of immunosuppression are similar in many points.

For the first time in France, we studied the QoL of a large sample of transplanted children including liver, kidney, and heart transplanted children, and their parents. We compared, using well-validated self-reported questionnaires, their QoL with that of samples of individuals presenting other various health conditions. The objectives of our study were: (1) to assess the QoL levels of the transplanted children and their parents and to compare it with those of individuals with other chronic conditions associated with long-term consequences (childhood leukaemia survivors and children with inborn errors of metabolism), and (2) to assess the potential factors modulating the QoL of children and their parents.

## Methods

### Study design and population

This study incorporated a cross-sectional design performed at the multidisciplinary paediatric centre of a French public teaching hospital (La Timone, Marseille, France). Children and their parents were included. The inclusion criteria were as follows: (1) for children: child with a history of organ transplantion (liver, kidney, or heart) transplanted for more than 1 year and less than 10 years, born between 1998 and 2011, with parents or legal guardians authorizing participation in the study; and (2) for parents: parents of a predefined child. A medical database allowed the identification of eligible children according to the selection criteria. The study was proposed to consecutive parents and children during a planned routine visit between June and November 2015.

### Ethical aspects

The protocol was approved by the ethics committee of Aix-Marseille University, France (reference number: 2014-08-04-03). According to French law (Article L1121-1, Law no. 2011-2012 29 December 2011, art. 5), all children and parents were fully informed of the study. This study was conducted in accordance with the Declaration of Helsinki and French Good Clinical Practices. Written consent was collected for each included parent.

### Medical records

For the children, the following data were collected: (1) sociodemographic: sex and age of the child and grade retention; (2) clinical data: the nature of the transplanted organ (liver, kidney, or heart), the age at transplantation, the time since transplantation, the occurrence of a transplant rejection (biopsy), post-transplantation radiointervention or surgery, background treatment, regular treatment (treatment other than the immunosuppressive therapy), immunosuppressive medication switch, latest residual treatment level (satisfactory, unsatisfactory), and the number of hospitalizations after transplantation.

For the parents the following sociodemographic data were collected: age, gender (mother or father), marital status (single, couple), and professional status (worker, non-worker). The number of siblings was also recorded.

### Evaluation of quality of life

#### Children

The QoL of the children and adolescents was assessed using a structured standardized questionnaire named the Vécu et Santé Perçue de l’Adolescent et de l’Enfant (VSP-A) [21, 22]. The parent version, VSP-Ap, is designed to be answered by the parents of children or adolescents of all ages (from 4 to 18 years). The 37 items describe 10 dimensions: relations with parents (RPa), body image (BI), vitality (VIT), relations with friends (RFr), leisure activities (LEI), psychological well-being (PsWB), physical well-being (PhWB), school performance (SCH); relations with teachers (RT), and relations with medical staff (RMS). All scores range between 0 and 100, with higher scores indicating a better QoL. Two child versions (VSP-Ac for children aged 8–10 years and VSP-At for teenagers aged 11–17 years) and one parent version are available. In the two child versions, 7 dimensions were common (VSP-A): relations with parents/family (RFa), body image/self-esteem (BI), vitality (VIT), relations with friends (RFr), leisure activities (LEI), school performance (SCH), and relations with medical staff (RMS). The scores of children with chronic conditions associated with long-term consequences are also available from previous studies coordinated by our team: childhood leukaemia survivor children [23] and children with inborn errors of metabolism with restricted diet [24]. French norms are not yet available.

#### Parents

Parents’ QoL was assessed using the French version of the World Health Organization Quality of Life (WhoQoL-BREF) questionnaire, which is a generic questionnaire of 26 items used worldwide [25] that describes four domains: physical health, psychological health, social relationships, and environment. French norms are available only for three domains: physical health, psychological health, and social relationships [26]. The scores of parents of children with inborn errors of metabolism with restricted diet are also available [24].

### Statistical analysis

Continuous variables were expressed as the means and standard deviations or the medians and interquartile ranges (IQR). Qualitative variables were expressed as numbers and percentages. Nonparametric statistics were used. The VSPA-p scores were compared with the scores obtained from French parents of children suffering from inborn errors of metabolism with restricted diet [24] and French parents of childhood leukemia survivors [23]. The VSP-A scores of the children and adolescents were compared to the scores obtained from French childhood leukaemia survivors [23]. The WhoQoL scores of the parents were compared with the scores obtained from French parents of children suffering from of inborn errors of metabolism with restricted diet [24] and from French age-sex-crossed norms [26]. Comparisons of mean QoL scores between different subgroups were performed using the Mann–Whitney tests for qualitative variables and Spearman’s correlation coefficients for quantitative variables. The statistical analyses were performed using the SPSS software package, version 20.0 (SPSS Inc., Chicago, IL, USA). All tests were two-sided. Statistical significance was defined as p < 0.05.

## Results

**Fig. 1:**
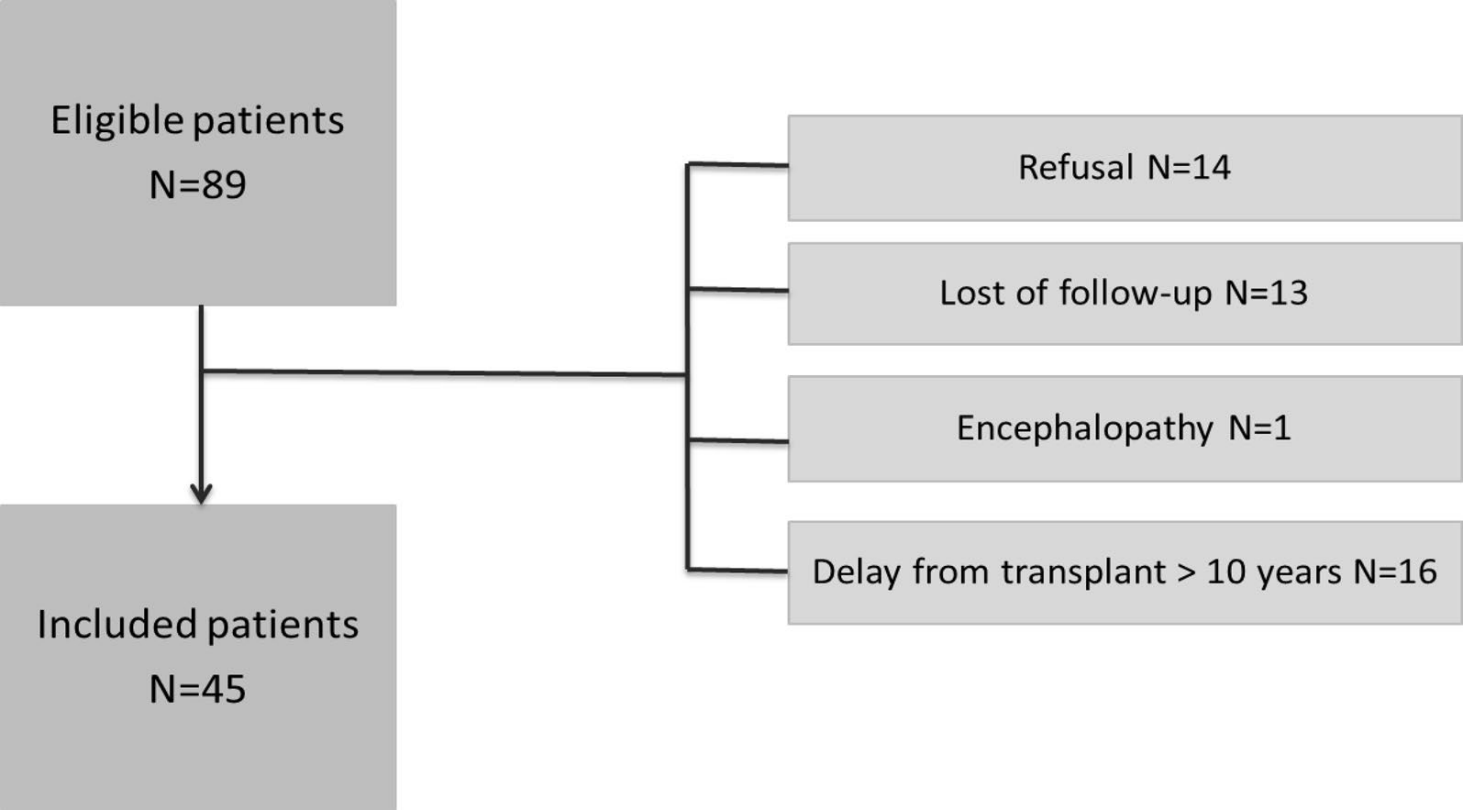
Chart

Among 89 eligible families, 30 met an exclusion criterion and 14 families did not participate. Forty-five patients were included leading to a response rate of 76%. The respondents and non-respondents did not differ according to the nature of the transplanted organ, age, and sex. Among the 45 patients, twenty children received a liver transplant, 15 received a kidney transplant, and 10 received a heart transplant. The median age at the time of the study was 9 years [IQR 6–12] and that at transplantation was 54 months [IQR 22–91]. The median time since transplantation was 52 [IQR 29–75] months. Nineteen children had a transplant rejection, 16 had a reoperation, and 28 had a radio-interventional procedure. At the evaluation time, 9 children had a residual level of treatment not in the target range, 28 had an immunosuppressive medication switch and 28 had a treatment other than the immunosuppressive therapy (regular treatment). Fourteen children presented a school delay greater than 1 year (Fig. 1).

The participating parents were mothers for 73% of patients with a median age of 42 [IQR 38–47] years. Parents were in couples in 78% of cases. All characteristics are detailed in Table 1.

**Table 1:** Participants characteristics

1. Children	N = 45
	N (%) or med [IQR]
*Gender*	
Girls	19 (42.2)
Boys	26 (57.8)
Age (years)	9.3 [5.6–12.4]
*School level*	
Not schooled	2 (4.4)
Appropriate level	29 (64.4)
Grade retention*	14 (31.1)
*Siblings*	
No	4 (9.1)
Yes	40 (90.9)
Number	2.0 [1.0–2.0]
*Transplant organ*	
Liver	20 (44.4)
Kidney	15 (33.3)
Heart	10 (22.2)
Age at transplant (months)	53.8 [21.7–90.6]
Delay from the transplant (months)	51.7 [28.6–74.6]
*Graft rejection*	
Yes	19 (43.2)
No	25 (56.8)
*Reject type*	
Acute rejection	15 (83.3)
Chronic rejection	2 (11.1)
*Reoperation*	
Yes	16 (36.4)
No	28 (63.6)
*Radio-interventionel procedure*	
Yes	28 (65.1)
No	15 (34.9)
Total number of medications	3.0 [2.0–5.0]
*Number of immunosuppresive drugs*	
1	17 (39.5)
≥2	26 (60.5)
*Residual treatment level*	
Satisfactory	31 (77.5)
Not satisfactory	9 (22.5)
*Immunosuppressive medication switch*	
Yes	28 (65.1)
No	15 (34.9)
Regular treatment**	
Yes	28 (65.1)
No	15 (34.9)

2. Parents	N = 45
	N (%) or med [IQR]
Mother	33 (80.5)
Father	8 (19.5)
Age (years)	42.0 [38.0–46.8]
*Marital status*	
Single	9 (20.5)
Couple	35 (79.5)
*Educational level*	
< 12 years	22 (52.4)
≥ 12 years	20 (47.6)
*Professional status*	
Worker***	25 (56.8)
Non-workers	19 (43.2)

Med [IQR], median [interquartile range]

*Grade retention defined as 1 year retention

**Other treatment associated with immunosuppressive drug

***At least one of the 2 parents

### Quality of life of transplanted children compared with that of other populations

#### Quality of life of children reported by the parents

**Fig. 2:**
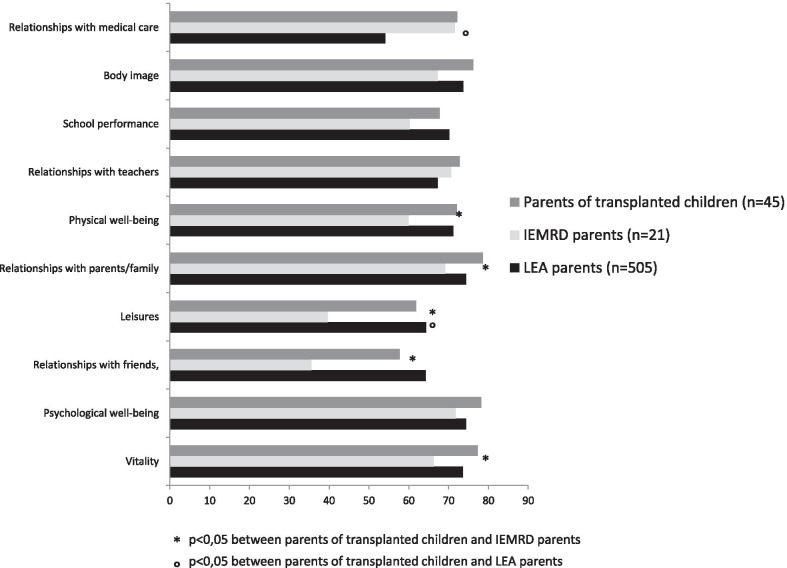
Quality of life of transplanted children reported by their parents

The VSP-Ap scores of transplanted children did not differ from those of childhood leukaemia survivors, except for scores for leisure activities, where childhood leukaemia survivors reported significantly higher scores and scores for relationships with medical care providers, which were lower than those of childhood leukaemia survivors. In contrast, compared to children suffering from inborn errors of metabolism with restricted diet, the QoL scores of transplanted children were significantly better for leisure activities, relationships with friends, vitality, and relationships with family. The details are presented in Fig. 2.

#### Self-reported quality of life of children

We observed that the QoL levels of transplanted young children (6–10 years) were: (1) higher than the QoL levels of children suffering from inborn errors of metabolism with restricted diet, except for school performance and leisure activities; and (2) lower than the QoL levels of childhood leukaemia survivors, except for vitality. In the same way, we saw that the QoL levels of transplanted teenagers (11–18 years) were: (1) higher than the QoL levels of children suffering from inborn errors of metabolism with restricted diet, except for 2 of the 9 dimensions (relationships with teachers and relationships with family); and (2) higher than the QoL levels of childhood leukaemia survivors for 7 of the 9 dimensions. Because of a limited number of cases (only 18 children aged from 6 to 10 years and 12 teenagers aged from 11 to 18 answered the VSP-A), we did not perform statistics to compare QoL levels with other populations. All the details are provided in the Additional file 1: Files 1 and 2).

#### Quality of life of parents

**Fig. 3:**
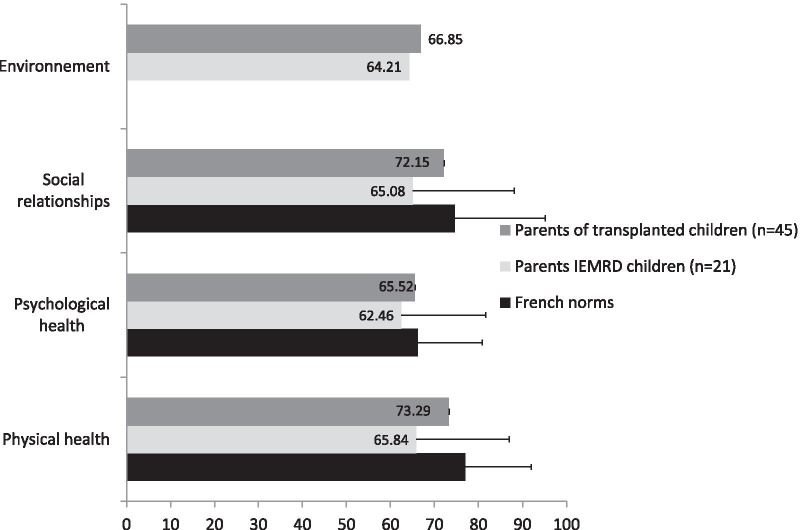
Quality of life of parents of transplanted children. Comparisons of WhoQoL scores between the parents of transplanted children and IEMRD parents and French age-sex-crossed norms

The QoL of parents of transplanted children did not differ from that of parents of children suffering from inborn errors of metabolism with restricted diet and from that of French age-sex-crossed norms. All the details are provided in Fig. 3.

### Variables modulating the quality of life

#### Quality of life of transplanted children reported by their parents

The factors significantly associated with QoL scores were as follows: (1) older children at the time of study reported better relationships with teachers than younger children; (2) the body image score was lower for older children at the time of the study and at transplant, for children of parents with a lower educational level, and for children with more siblings; (3) relationships with friends and leisure activities were better for children of parents with a job; (4) radiointervention was associated with a lower school score; (5) QoL scores did not differ according to the nature of the transplanted organ, except for the vitality score which was lower for children with a kidney transplant; (6) the occurence of a medication switch in immunosuppression decreased scores for leisure activities and school performances; (7) an unsatisfactory residual treatment level was associated with better scores for relationship with family and vitality. Sex of the child, grade retention, parental marital status, parental age, time since transplantation, rejection, and reoperation were not associated to QoL. All the results are provided in Table 2.

**Table 2:** Factors modulating children quality of life reported by parents (VSP-Ap): N=45

	VIT	PsWB	RFr	LEI	RPa	PhWB	RT	SCH	BI	RMS
*Gender of the child*										
Boys	80.1 ± 13.8	77.6 ± 20.1	56.8 ± 29.4	65.0 ± 57.0	79.0 ± 13.2	70.3 ± 22.0	76.1 ± 24.8	69.8 ± 15.2	76.3 ± 16.5	75.4 ± 31.9
Girls	73.5 ± 16.0	79.0 ± 15.7	59.0 ± 21.6	57.7 ± 22.0	78.1 ± 14.2	74.7 ± 19.7	68.4 ± 33.5	64.8 ± 18.9	76.1 ± 13.2	68.1 ± 32.7
p value	NS	NS	NS	NS	NS	NS	NS	NS	NS	NS
Age of the child (R)	− 0.243	− 0.146	0.139	− 0.008	− 0.070	− 0.008	− 0.353	− 0.236	− 0.467	0.053
p value	NS	NS	NS	NS	NS	NS	**0.030**	NS	0.002	NS
*Grade retention*										
No	78.0 ± 14.6	77.5 ± 19.6	58.5 ± 24.5	63.6 ± 53.2	79.6 ± 12.8	73.1 ± 20.6	78.5 ± 23.0	71.0 ± 15.2	78.2 ± 14.7	65.7 ± 36.2
Yes	75.4 ± 16.9	78.1 ± 17.0	55.8 ± 31.0	54.4 ± 25.5	77.5 ± 15.6	69.6 ± 21.6	66.3 ± 35.1	61.5 ± 18.0	70.2 ± 14.7	83.3 ± 19.5
p value	NS	NS	NS	NS	NS	NS	NS	NS	NS	NS
Age of parent (R^a^)	− 0.194	− 0.165	0.099	0.065	− 0.163	− 0.147	− 0.248	− 0.188	− 0.224	0.076
p value	NS	NS	NS	NS	NS	NS	NS	NS	NS	NS
*Parental marital status*										
Single	76.9 ± 21.7	76.8 ± 29.0	49.2 ± 32.2	49.4 ± 28.6	79.4 ± 21.4	70.8 ± 25.8	70.8 ± 33.3	66.7 ± 16.5	77.1 ± 17.5	67.9 ± 29.0
Couple	77.5 ± 13.0	78.3 ± 16.1	59.9 ± 24.4	65.0 ± 48.2	78.3 ± 11.1	72.1 ± 20.2	73.2 ± 28.6	68.1 ± 17.0	75.3 ± 14.3	73.2 ± 33.0
p value	NS	NS	NS	NS	NS	NS	NS	NS	NS	NS
*Parent’ educational level*										
< 12 years	75.2 ± 17.9	76.0 ± 22.1	53.6 ± 26.4	55.2 ± 24.2	78.0 ± 15.0	70.7 ± 23.9	69.5 ± 33.6	68.2 ± 14.3	71.0 ± 14.5	80.8 ± 23.4
≥ 12 years	80.0 ± 11.2	80.6 ± 14.5	61.4 ± 26.4	69.6 ± 61.9	78.5 ± 12.3	73.8 ± 18.4	74.5 ± 24.5	68.4 ± 19.8	80.2 ± 14.5	61.1 ± 37.6
p value	NS	NS	NS	NS	NS	NS	NS	NS	**0.047**	NS
*Parent’ professional status*										
Workers	77.8 ± 13.9	79.4 ± 16.3	67.4 ± 20.1	71.9 ± 53.8	81.8 ± 10.7	70.6 ± 17.3	79.0 ± 24.5	68.5 ± 17.6	78.5 ± 12.7	67.0 ± 37.9
Not workers	76.7 ± 16.5	76.0 ± 21.5	44.6 ± 28.0	48.7 ± 25.7	74.3 ± 16.1	73.4 ± 25.6	64.1 ± 33.4	66.9 ± 15.9	71.7 ± 16.8	79.7 ± 19.7
p value	NS	NS	**0.014**	**0.047**	NS	NS	NS	NS	NS	NS
Siblings number (R^a^)	− 0.301	− 0.154	− 0.141	− 0.124	− 0.159	0.005	− 0.265	− 0.162	− **0.339**	0.055
	NS	NS	NS	NS	NS	NS	NS	NS	0.035	NS
*Transplant*										
Liver	84.9 ± 13.3	84.1 ± 18.1	61.5 ± 27.6	55.7 ± 19.0	80.3 ± 15.2	73.0 ± 21.5	83.3 ± 24.2	70.6 ± 16.5	80.7 ± 14.3	70.4 ± 34.0
Kidney	70.8 ± 11.2	74.9 ± 16.1	52.1 ± 25.1	70.5 ± 72.9	79.2 ± 10.6	72.1 ± 19.7	59.8 ± 34.5	61.6 ± 17.3	69.9 ± 11.9	78.5 ± 27.6
Heart	72.2 ± 17.6	73.1 ± 20.5	60.6 ± 26.0	61.1 ± 22.5	74.8 ± 14.6	70.2 ± 23.8	75.0 ± 20.4	72.2 ± 15.0	77.1 ± 18.5	67.6 ± 35.7
p value	**0.003**	NS	NS	NS	NS	NS	NS	NS	NS	NS
Age at transplant (R^a^)	− 0.199	− 0.157	0.140	0.158	− 0.147	− 0.024	− 0.219	− 0.191	− 0.519	0.158
p value	NS	NS	NS	NS	NS	NS	NS	NS	**<0.001**	NS
Delay from transplant (R^a^)	− 0.112	− 0.036	0.064	− 0.238	0.086	− 0.004	− 0.203	− 0.110	− 0.032	− 0.014
p value	NS	NS	NS	NS	NS	NS	NS	NS	0.035	NS
*Graft rejection*										
Yes	75.2 ± 15.1	76.4 ± 17.4	53.8 ± 29.3	49.4 ± 19.8	78.1 ± 15.0	72.8 ± 17.5	68.4 ± 25.6	66.4 ± 18.1	76.1 ± 16.7	78.6 ± 28.0
No	78.4 ± 15.1	79.0 ± 19.4	59.5 ± 23.6	70.1 ± 56.2	78.9 ± 12.7	70.8 ± 23.9	76.2 ± 32.0	69.0 ± 16.4	76.1 ± 14.4	66.7 ± 34.8
p value	NS	NS	NS	NS	NS	NS	NS	NS	NS	
*Reoperation*										
Yes	78.1 ± 16.5	79.5 ± 19.6	67.0 ± 24.5	61.3 ± 22.1	82.0 ± 13.1	69.1 ± 21.2	79.8 ± 30.8	67.5 ± 16.9	76.9 ± 11.9	60.0 ± 37.8
No	77.4 ± 14.3	76.9 ± 17.9	54.5 ± 25.0	63.7 ± 54.8	77.0 ± 13.6	73.5 ± 21.4	68.8 ± 27.5	67.7 ± 17.3	76.1 ± 16.8	79.3 ± 26.2
p value	NS	NS	NS	NS	NS	NS	NS	NS	NS	NS
*Radiointervention*										
Yes	75.0 ± 15.9	75.2 ± 19.8	60.5 ± 27.3	55.8 ± 23.3	79.2 ± 13.8	69.3 ± 23.1	68.9 ± 31.1	63.0 ± 15.8	73.3 ± 15.8	69.1 ± 32.9
No	83.3 ± 12.2	83.5 ± 14.9	55.3 ± 22.3	76.7 ± 69.1	79.2 ± 13.5	77.7 ± 16.8	82.6 ± 19.9	76.8 ± 15.4	82.7 ± 12.5	74.4 ± 32.4
p value	NS	NS	NS	NS	NS	NS	NS	**0.020**	NS	NS
*Last residual treatment level*										
Satisfactory	73.9 ± 13.9	79.7 ± 18.2	57.7 ± 25.9	66.2 ± 52.4	77.2 ± 10.6	71.7 ± 23.1	67.7 ± 30.2	67.6 ± 16.4	76.0 ± 15.1	74.1 ± 32.1
Not satisfactory	87.6 ± 13.1	83.3 ± 13.5	64.3 ± 28.8	57.2 ± 22.5	86.8 ± 12.1	77.1 ± 18.2	88.1 ± 22.5	69.4 ± 17.8	79.6 ± 17.2	65.5 ± 39.2
p value	**0.025**	NS	NS	NS	**0.037**	NS	NS	NS	NS	NS
*Medication switch*										
Yes	77.3 ± 14.8	79.5 ± 19.2	57.7 ± 25.6	60.6 ± 54.9	78.0 ± 13.7	74.6 ± 20.9	67.9 ± 30.6	64.4 ± 16.2	76.5 ± 12.9	73.4 ± 32.5
No	77.6 ± 16.3	75.5 ± 18.0	62.2 ± 23.2	66.9 ± 16.4	78.8 ± 14.1	64.3 ± 20.0	84.0 ± 23.7	75.0 ± 16.1	76.1 ± 16.6	69.9 ± 32.2
p value	NS	NS	NS	**0.049**	NS	NS	0.08	**0.043**	NS	NS
*Regular treatment*										
Yes	76.0 ± 16.6	76.0 ± 21.3	55.6 ± 28.0	54.0 ± 23.1	78.7 ± 14.7	71.1 ± 24.0	74.5 ± 29.8	66.7 ± 18.3	73.8 ± 15.5	72.2 ± 33.0
No	79.4 ± 12.1	81.8 ± 10.8	61.3 ± 23.0	76.9 ± 69.5	76.8 ± 11.0	73.3 ± 16.4	67.3 ± 28.8	69.2 ± 14.8	79.2 ± 13.1	72.2 ± 31.4
p value	NS	NS	NS	NS	NS	NS	NS	NS	NS	NS

VIT, vitality; PsWB, psychological well-being; RFr, relations with friends; LEI, leisure activities; RPa, relations with parents; PhWB, physical well-being; RT, relations with teachers; SCH, school performance; BI, body image; RMS, relations with medical staff

NS: non significant; R: correlation coefficient; Bold values: p values < 0.05; higher scores indicate higher QoL

^*^Number of hospitalizations after transplant

#### Self-reported quality of life of transplanted children

The scores for the 7 common dimensions between the child version and the teenager version were available for 28 individuals. The factors associated with QoL scores were as follows: (1) older children at the time of the study had better scores for relationships with friends and school performance; (2) the absence of grade retention was associated with a better score relationship with friends; (3) older parents had better scores for relationships with family; (4) when the 2 parents were a couple, the scores for relationships with medical care providers were significantly better; (5) children with more siblings had better scores for body image; (6) older children at transplantation had better scores for relationships with friends; (7) children with longer times since transplantation had higher school performance scores; (8) the occurrence of a medication switch was associated with lower leisure activities scores; (9) children with no regular treatment had higher body image scores. The QoL scores did not differ according to the gender of the child, parental education level and professional status, the nature of the transplanted organ, rejection/reoperation/radiointervention and residual treatment level. All the results are detailed in Table 3.

**Table 3:** Factors modulating self-reported QoL of children (VSP-A): N=28

	RFa	BI	VIT	RFr	LEI	SCH	RMS
*Gender of the child*							
Boys	70.4 ± 15.3	77.5 ± 18.5	82.7 ± 20.2	53.8 ± 31.8	60.7 ± 20.1	70.8 ± 19.2	67.1 ± 29.6
Girls	59.8 ± 20.4	74.5 ± 20.0	74.4 ± 23.1	58.2 ± 24.2	55.1 ± 26.6	70.8 ± 19.8	78.1 ± 34.2
p value	NS	NS	NS	NS	NS	NS	NS
Age of the child (R)	− 0.090	0.265	− 0.150	0.491	− 0.163	0.443	− 0.073
p value	NS	NS	NS	**0.009**	NS	**0.024**	NS
*Grade retention*							
No	74.1 ± 12.3	79.6 ± 17.3	82.8 ± 18.9	60.6 ± 26.9	58.4 ± 19.0	73.4 ± 17.0	68.2 ± 31.9
Yes	57.3 ± 20.3	72.0 ± 21.4	74.2 ± 24.1	46.6 ± 32.8	56.8 ± 25.8	67.5 ± 23.0	73.1 ± 31.9
p value	**0.020**	NS	NS	NS	NS	NS	NS
Age of parent (R)	0.100	0.113	− 0.018	**0.435**	− 0.054	0.371	0.135
p value	NS	NS	NS	**0.021**	NS	NS	NS
*Parental marital status*							
Single	61.3 ± 13.9	64.3 ± 23.2	66.7 ± 33.1	45.4 ± 27.0	53.5 ± 32.3	64.6 ± 14.6	36.1 ± 28.2
Couple	68.6 ± 18.3	79.9 ± 16.3	83.7 ± 15.7	57.8 ± 29.7	60.3 ± 19.1	72.6 ± 20.0	80.8 ± 23.6
p value	NS	NS	NS	NS	NS	NS	**0.001**
*Parent’ educational level*							
< 12 years	62.9 ± 20.1	74.4 ± 21.8	77.1 ± 23.0	49.7 ± 31.0	58.4 ± 25.5	66.1 ± 23.7	66.0 ± 33.8
≥ 12 years	71.7 ± 13.1	78.8 ± 15.6	83.5 ± 19.1	61.5 ± 26.6	59.4 ± 18.3	76.0 ± 10.8	75.0 ± 28.3
p value	NS	NS	NS	NS	NS	NS	NS
*Parent’ professional status*							
Workers	71.0 ± 16.1	78.3 ± 19.6	80.3 ± 19.7	59.2 ± 29.4	61.5 ± 18.8	75.7 ± 17.4	71.9 ± 31.6
Not workers	59.9 ± 18.5	73.9 ± 17.9	79.5 ± 24.7	48.0 ± 28.7	54.1 ± 27.4	62.5 ± 19.5	68.3 ± 31.1
p value	NS	NS	NS	NS	NS	NS	NS
Siblings number (R^a^)	0.004	0.483	0.131	− 0.216	0.210	− 0.119	0.246
p value	NS	**0.014**	NS	NS	NS	NS	NS
*Nature of the transplant*							
Liver	71.1 ± 12.1	78.6 ± 18.6	82.4 ± 21.0	53.1 ± 35.4	60.5 ± 22.6	71.3 ± 17.7	63.3 ± 37.5
Kidney	65.7 ± 20.8	78.8 ± 17.1	82.5 ± 24.6	56.1 ± 27.9	64.1 ± 18.0	73.8 ± 16.1	81.7 ± 19.6
Heart	62.5 ± 20.6	70.2 ± 22.5	72.9 ± 16.8	57.1 ± 23.6	48.9 ± 26.2	66.1 ± 25.7	63.9 ± 33.6
p value	NS	NS	NS	NS	NS	NS	NS
Age at transplant (R)	0.072	0.161	− 0.060	0.430	0.023	0.225	− 0.005
p value	NS	NS	NS	**0.025**	NS	NS	NS
Delay from transplant	− 0.021	0.071	− 0.151	0.249	− 0.230	**0**.415	− 0.009
p value	NS	NS	NS	NS	NS	**0.031**	NS
*Graft rejection*							
Yes	70.0 ± 20.5	74.7 ± 20.9	80.4 ± 21.0	54.5 ± 36.3	54.2 ± 24.2	70.8 ± 22.8	75.0 ± 28.9
No	64.8 ± 15.6	78.2 ± 17.1	79.7 ± 22.6	54.3 ± 24.3	63.6 ± 20.5	70.8 ± 16.1	66.7 ± 33.0
p value	NS	NS	NS	NS	NS	NS	NS
*Reoperation*							
Yes	68.2 ± 10.5	83.1 ± 15.7	82.6 ± 22.1	54.6 ± 29.0	65.0 ± 22.7	75.0 ± 8.83	60.2 ± 37.9
No	66.3 ± 20.6	73.5 ± 19.6	78.6 ± 21.1	55.5 ± 30.1	55.5 ± 21.6	68.8 ± 22.4	76.0 ± 26.0
p value	NS	NS	NS	NS	NS	NS	NS
*Radiointervention*							
Yes	64.0 ± 19.2	72.9 ± 18.4	79.2 ± 21.6	52.6 ± 31.3	54.2 ± 23.0	66.2 ± 21.5	77.6 ± 27.2
No	72.1 ± 13.7	81.3 ± 18.5	79.4 ± 21.6	56.5 ± 24.9	69.9 ± 17.6	77.8 ± 10.4	54.6 ± 33.1
p value	NS	NS	NS	NS	NS	NS	NS
*Last residual treatment level*							
Satisfactory	66.7 ± 17.7	80.5 ± 17.5	81.4 ± 18.5	54.9 ± 29.6	60.2 ± 20.8	75.0 ± 16.6	72.5 ± 25.1
Not satisfactory	68.4 ± 12.8	71.8 ± 18.2	81.4 ± 26.6	53.8 ± 34.3	63.0 ± 20.1	62.5 ± 20.4	71.4 ± 36.0
p value	NS	NS	NS	NS	NS	NS	NS
*Medication switch*							
Yes	66.0 ± 17.0	78.9 ± 19.0	79.3 ± 20.7	49.9 ± 31.4	53.1 ± 22.0	73.0 ± 13.3	69.9 ± 30.9
No	69.5 ± 19.6	69.6 ± 18.4	81.9 ± 23.4	68.4 ± 17.9	73.3 ± 15.0	65.6 ± 28.9	71.9 ± 32.7
p value	NS	NS	NS	NS	**0.038**	NS	NS
*Regular treatment*							
Yes	66.2 ± 18.0	70.0 ± 19.3	75.1 ± 23.7	58.3 ± 29.9	54.7 ± 24.0	68.4 ± 23.0	70.3 ± 31.0
No	68.5 ± 17.4	88.3 ± 10.4	89.0 ± 12.0	49.6 ± 28.5	66.4 ± 16.5	75.0 ± 8.3	70.8 ± 32.2
p value	NS	**0.016**	NS	NS	NS	NS	NS

RFa, relations with parents/family; BI, body image/self-esteem; VIT, vitality; RFr, relations with friends; LEI, leisures; SCH, school performance; RMS, relations with medical staff; higher scores indicate higher QoL

Bold values: p values< 0.05

^a^R: correlation’s coefficients

#### Quality of life of parents of transplanted children

An unsatisfactory residual treatment level was associated with a better quality of life in the physical dimension. No other variable was associated with parents QoL. All the results are detailed in Additional file 1: File 3.

## Discussion

We have studied the quality of life and the modulating factors of a sample of 45 transplanted children and their parents, including, for the first time, liver, kidney, and heart transplanted children together.

A first interesting finding is that the QoL of transplanted children (as reported by their parents or by themselves) did not differ regarding the organ type. Two other studies [27, 28] did not find significant differences between liver and kidney transplant recipients. Most likely after transplantation, the specificity of the organ becomes less important, and daily life becomes similar to that of individuals with another chronic condition. The immunosuppressive drugs and follow-up are almost the same for these three organ transplantations. This similar QoL may also be explained by the specificity of the centre where the study was conducted. After the transplantation procedure, all children and families are managed in the same care unit by the same team. This unit offers medical, psychological, and social support in the same location, allowing care standardization and resource sharing. Scores for relationships with medical care providers, higher for the transplanted children than for childhood leukaemia survivors, suggest that this kind of organization satisfies the families. The multidisciplinary staff is trained to coordinate and optimize the care trajectory. Families may have access to familiar professionals that improve understanding and faith. Some common educational therapy workshops could be put into place to offer self-knowledge and support to children and their parents.

In our study, we compared our sample to children with other conditions: childhood leukaemia survivors and children suffering from inborn errors of metabolism. Children’s QoL reported by parents was close to the QoL reported by childhood leukaemia survivors, which had been described in other studies [19, 29], and was better than, for most dimensions, the QoL of children suffering from inborn errors of metabolism with restricted diet. Some hypotheses could be made. While the period around the transplantation process may be considered critical, after transplantation, everyday life progressively becomes close to a “normal life”. With time, the occurrence of severe, fatal and lethal events decreases, reducing emotional and physical impacts. The course of disease at this point of a transplanted individual looks similar to that of a person with acute leukaemia: daily life gradually normalizes as the person transitions out of the acute therapeutic period. Transplanted children do not heal, but the disease burden often decreases. In contrast, children suffering from inborn errors of metabolism with restricted diet are confronted daily and continuously with the disease and its consequences. A lethal risk is often present and leads to permanent stress. Limbers et al [19] demonstrated similar findings in a liver transplant cohort: QoL was better than that in children on renal dialysis, similar to that in renal transplantation patients and patients in cancer remission. Taylor et al [13], also in a cohort of liver transplant children, found that the QoL was similar to that of individuals with other chronic situations, such as asthma and diabetes.

Parents’ QoL did not differ from the QoL of parents of children suffering from inborn errors of metabolism with restricted diet, or, more surprisingly, from French (age-sex-crossed) norms. This finding could be partially explained by the presence of a well-known phenomenon: ‘response shift’ or ‘adaptation to illness’ or ‘coping’ [30]. Coping is commonly defined as the cognitive and behavioural efforts that are implemented to solve problems and to reduce the stress that these problems may cause. In many various chronic diseases [31–34], it has been shown that self-reported QoL is not associated with objective health status due to the ability of individuals to adapt to manage the realities life. Because they have known the diagnosis for several years or since their child’s birth, parents adapt themselves to the illness over time and thus report corresponding QoL. Due to a lack of reference we did not compare QoL of parents in our sample to that of parents of leukaemia survivors. In the future, it could also be interesting to study how patients and caregivers handle problems in daily life and their ability to cope with difficulties.

The last part of our findings refers to QoL determinants. The identification of QoL determinants may help to find unmet needs, prioritize service improvements, and support funding decisions. The analyses that we performed showed that the main sociodemographic and socioeconomic parameters (such as gender, parent’s marital status, and parent’s educational level) cannot be identified as significant QoL determinants. QoL scores were not associated with the nature of the transplant organ while the notion of medication switch or residual level range seemed to be more important modulators. This result may surprise. Indeed, because the consequence of a graft failure is different, we could have expected that the heart or liver recipients (and their caregivers) report worse QoL than kidney recipients. Graft failure is always a dramatic event for the heart-liver situations that needs to find a relevant donor on time while kidney recipients would be provided chronic dialysis. This lack of difference in our sample could be explained by the fact that we only included children transplanted for more than 1 year. At this time, the risk of graft failure is less important than close to the transplant date. These findings suggest that organ transplantation, whatever the nature of the organ, may be considered as a global and homogeneous chronic condition.

We only found ectopic associations, expected or not. Not surprisingly, older children (and similarly later transplanted children) reported better QoL levels in their relationship with friends and lower levels in their relationship with teachers, reflecting the expected relationships during the specific time of adolescence. Children with longer times since transplantation had better school scores, explained by the effect of the transplantation procedure on daily activities of the child including school. Radiointervention was associated with a lower school score. Health care teams should reinforce actions to disrupt school less. In their study on liver recipients, Alonso et al [35] found similar findings, with the occurrence of reoperations and diabetes post transplantation impacting the QoL.

Long-term therapeutic necessity appears common in these conditions, disrupting everyday life and free time. The necessity to change a treatment for intolerance or inefficacy, and the necessity of residual treatment level control are parameters that disturb the QoL. The occurrence of a medication switch decreased scores for leisure activities and school performance. Medications and several appointments can prevent children from eating lunch at school or from doing some outdoor school activities. Each change in the treatment requires several blood tests and consultations at the hospital. Health care providers must adapt their practice to the child and family, not the inverse. The fact that a residual of treatment not in the target is associated with better relationship between children and their family and parents’ physical well-being suggests that treatment that impacts daily life less, results in better QoL. Self-knowledge of signs and symptoms of transplant rejection or other complications should be learned by children and their families in order to consult their treatment providers early and to avoid heavy treatment or hospitalization.

### Strengths and limitations

One strength of the study is the nature of QoL questionnaire’s used. They have interesting characteristics in comparison with previous studies: (1) their content is based on patients’ or family’s point of view, known to provide more valid information than contents based on experts’ point of view [36]; (2) the validation process is based on a well-established procedure while previous studies used tools based on incomplete (or inappropriate) validation processes. Unfortunately, we could not compare the VSPA scores of our sample with French norms, due to the unavailability of these at this time.

Additionally, our study is one of the first to study parents’ QoL, although they are the primary caregivers for most children.

Another strength is that we assessed for the first time heart transplanted children. Only two studies provide data from French population [5, 14] with a kidney and liver transplant cohort. The transferability of findings of the non-French population is difficult because the QoL and satisfaction are closely dependent on cultural background and the health-care system. Free choice of health-care and universal health-care insurance are particular to France and lead to variations in patients’ and families’ expectations [37]. Almost all the health care system is free in France which might explain that why sociodemographic parameters do not impact the QoL.

One limitation of the study is our small sample size which did not allow for a multivariate approach. Potential confounding factors and moderate associations were not assessed (living-related transplantation for example) or possibly missed due to low power. The number of patients kidney transplants (n = 15) should be cautiously interpreted. The replication of these findings in larger groups of patients is required.

Due to the participation rate, the representativeness of our study could be questioned. We could hypothesize that the non-participants included families of children with more severe physical and/or mental conditions, which would have led to a global overestimation of the quality of life. However, the respondents did not differ from the non-respondents in terms of the main characteristics (sociodemographic and clinical), which ensures the relative validity of our findings. Fewer liver transplant children were included in comparison with other organ transplant children, but this can be explained by the fact that this transplant requires fewer hospital visits and children were seen less during the inclusion period.

In this study, we compared our sample to children with other conditions: childhood leukaemia survivors and children suffering from inborn errors of metabolism. While these results are informative, other comparisons (normal population or individuals with milder pathology) should bring complementary information. Future studies should provide these findings.

The last limitation is the type of study which is cross-sectional. Cross-sectional studies examine individuals with heterogeneous disease durations. Longitudinal studies provide more valid information and are necessary to more precisely determine the weights of potential predictive factors of the quality of life. Future studies based on longitudinal cohorts will help to better understand families’ functioning.

## Conclusion

Children and their families reported a fairly preserved quality of life in comparison with those with other chronic health conditions. While the nature of the transplanted organ was not identified as a QoL modulator, special attention should be given to therapeutic management which might be amenable and is expected to improve the QoL.

## Supplementary Information

**Additional file 1**. Children’ QoL (6-10 years): comparisons of VSPA scores between the transplanted children and LEA children and IEMRD children.
**Additional file 2**. Teenagers’ QoL (11-18 years): comparisons of VSP-Ae scores between the transplanted children and LEA children and IEMRD children.
**Additional file 3**. Factor modulating parents QoL (WhoQoL): N=45.

## Data Availability

The datasets used and/or analyzed during the current study are available from the corresponding author on reasonable request.
