# Supplementary material for: Quality of life of transplanted children and their parents: a cross-sectional study
Source: Orphanet J Rare Dis. 2021 Aug 17;16:364. doi: 10.1186/s13023-021-01987-y (PMC8369793; doi:10.1186/s13023-021-01987-y)
Supplement: Supplementary file 1 — Additional file 1. Children’ QoL (6-10 years): comparisons of VSPA scores between the transplanted children and LEA children and IEMRD children. Additional file 2. Teenagers’ QoL (11-18 years): comparisons of VSP-Ae scores between the transplanted children and LEA children and IEMRD children. Additional file 3. Factor modulating parents QoL (WhoQoL): N=45. [file 13023_2021_1987_MOESM1_ESM.docx]

**Additional file 1. Children’ QoL (6-10 years): comparisons of VSPA scores between the transplanted children and LEA children and IEMRD children**

**Additional file 2. Teenagers’ QoL (11-18 years): comparisons of VSP-Ae scores between the transplanted children and LEA children and IEMRD children**

**Additional file 3. Factor modulating parents QoL (WhoQoL): N=45**

|  | **Physical** | **Psychological** | **Social** | **Environment** |
| --- | --- | --- | --- | --- |
| Gender of the child | |  |  |  |
| Boys | 74,2±13,7 | 64,9±16,0 | 70,5±18,8 | 65,0±16,6 |
| Girls | 71,9±17,0 | 66,5±12,9 | 74,3±23,0 | 69,5±18,2 |
| p-value | NS | NS | NS | NS |
| Age of the child° | -0,012 | -0,069 | -0,110 | -0,045 |
| p-value | NS | NS | NS | NS |
| Grade retention |  |  |  |  |
| No | 76,0±13,4 | 64,8±15,9 | 72,2±19,0 | 68,4±15,7 |
| Yes | 70,1±15,1 | 65,4±12,6 | 70,1±25,2 | 61,2±19,5 |
| p-value | NS | NS | NS | NS |
| Gender of parent |  |  |  |  |
| Mother | 74,3±13,2 | 66,8±11,9 | 74,6±13,8 | 68,1±16,8 |
| Father | 66,6±21,2 | 70,2±13,9 | 68,2±33,9 | 60,3±21,5 |
| p-value | NS | NS | NS | NS |
| Age of parent° | 0,010 | 0,102 | -0,196 | 0,056 |
| p-value | NS | NS | NS | NS |
| Parental marital status | |  |  |  |
| Single | 74,3±20,5 | 59,7±22,7 | 63,0±32,5 | 60,9±20,9 |
| Couple | 72,7±13,9 | 67,4±11,8 | 75,5±15,1 | 68,4±16,4 |
| p-value | NS | NS | NS | NS |
| Parent’ educational level |  |  |  |  |
| <12 y | 74,6±15,8 | 64,4±17,9 | 73,1±22,8 | 66,1±17,2 |
| >=12 y | 71,9±14,6 | 66,9±10,9 | 70,6±18,2 | 68,5±18,0 |
| p-value | NS | NS | NS | NS |
| Parent’ professional status |  |  |  |  |
| Workers | 74,3±12,9 | 67,2±12,2 | 75,5±15,1 | 70,7±16,2 |
| Not workers | 71,3±17,6 | 64,0±17,7 | 68,9±26,1 | 62,1±18,2 |
| p-value | NS | NS | NS | NS |
| Siblings number° | -0,097 | 0,016 | 0,112 | -0,210 |
| p-value | NS | NS | NS | NS |
| Nature of the transplant |  |  |  |  |
| Liver | 77,3±15,3 | 63,2±18,3 | 69,7±22,4 | 68,4±15,3 |
| Kidney | 71,9±10,9 | 69,9±10,9 | 77,2±6,5 | 66,9±18,5 |
| Heart | 68,2±19,1 | 63,2±11,5 | 70,0±28,7 | 63,7±20,1 |
| p-value | NS | NS | NS | NS |
| Age at transplant° | 0,028 | 0,075 | 0,083 | -0,093 |
| p-value | NS | NS | NS | NS |
| Delay from transplant° | -0,044 | -0,070 | -0,298 | 0,067 |
| p-value | NS | NS | NS | NS |
| Graft rejection |  |  |  |  |
| Yes | 71,0±17,4 | 67,1±11,9 | 67,6±20,4 | 63,7±18,4 |
| No | 74,8±13,2 | 65,0±16,5 | 75,0±20,5 | 68,6±16,2 |
| p-value | NS | NS | NS | NS |
| Last residual rate |  |  |  |  |
| Satisfactory | **71,9±14,4** | 64,6±15,7 | 74,2±18,6 | 66,6±17,3 |
| Not satisfactory | **83,6±7,00** | 69,9±10,0 | 72,7±16,5 | 71,4±13,9 |
| p-value | **0,032** | NS | NS | NS |
| Drug switch |  |  |  |  |
| Yes | 75,0±14,9 | 64,1±15,7 | 68,3±22,0 | 66,1±18,8 |
| No | 69,4±15,8 | 69,3±13,1 | 80,9±15,9 | 69,3±15,0 |
| p-value | NS | NS | NS | NS |
| Background treatment |  |  |  |  |
| Yes | 71,8±15,2 | 64,9±16,7 | 72,2±23,2 | 66,6±16,8 |
| No | 73,7±14,9 | 66,0±10,0 | 72,6±15,8 | 66,6±19,4 |
| p-value | NS | NS | NS | NS |
| Reoperation |  |  |  |  |
| Yes | 75,9±12,3 | 63,4±19,9 | 71,9±22,5 | 70,0±16,0 |
| No | 71,5±16,5 | 66,4±11,0 | 72,2±20,1 | 64,9±18,3 |
| p-value | NS | NS | NS | NS |
| Radio intervention |  |  |  |  |
| Yes | 70,6±15,4 | 64,8±15,9 | 70,8±22,1 | 66,2±18,7 |
| No | 78,6±14,1 | 65,7±13,2 | 75,3±19,0 | 67,8±16,0 |
| p-value | NS | NS | NS | NS |

NS: non significant; R: correlation coefficient; Bold values: p-values< 0.05; higher scores indicate higher QoL
